# Occurrence, Density, and Transcriptomic Response of the Leafhopper *Erythroneura sudra* (Hemiptera: Cicadellidae) When Confronted With Different Fruit Tree Species

**DOI:** 10.1093/jisesa/ieac037

**Published:** 2022-06-28

**Authors:** Yueyue Wang, Xunbing Huang, Hui Li, Guangyan Chen

**Affiliations:** Shandong Provincial Key Laboratory of Water and Soil Conservation and Environmental Protection, College of Resources and Environment, Linyi University, Linyi, P.R. China; Shandong Provincial Key Laboratory of Water and Soil Conservation and Environmental Protection, College of Resources and Environment, Linyi University, Linyi, P.R. China; Shandong Provincial Key Laboratory of Water and Soil Conservation and Environmental Protection, College of Resources and Environment, Linyi University, Linyi, P.R. China; Shandong Provincial Key Laboratory of Water and Soil Conservation and Environmental Protection, College of Resources and Environment, Linyi University, Linyi, P.R. China

**Keywords:** leafhopper, occurrence density, transcriptomics, host plants

## Abstract

The leafhopper, *Erythroneura sudra* (Distant) is becoming a dominant insect pest, and usually can cause significant damage to fruit production in northern China. We studied the occurrence and density of *E. sudra* on three fruit tree species and its transcriptomic responses when it was fed on leaves of these tree species. A higher density and survival rate of *E. sudra* were recorded when it fed on leaves of peach (*Amygdalus persica* L.) (Rosales: Rosaceae) and cherry (*Cerasus pseudocerasus* Lindl) (Rosales: Rosaceae) than on apple (*Malus domestica* Mill) (Rosales: Rosaceae). Also, feeding on *M. domestica* induced the largest variation in transcriptomic profiles in *E. sudra*. In total, 166 genes were differentially expressed (89 upregulated and 77 downregulated) in *E. sudra* when it fed on *M. domestica*, compared to when it fed on the other two tree species. The upregulated genes were mainly related to ‘response to oxidative stress’, ‘stress-resistance’, and ‘xenobiotic metabolic process’. The downregulated genes were mainly related to ‘structural constituent of cuticle’, ‘biosynthetic process’, and ‘development regulation’. These results suggested that *M. domestica* significantly changed the expression of many genes and consequently caused lower occurrence and density of *E. sudra*. Such information could enhance our understanding of the leafhopper–host plant relationship. Additionally, it can contribute to the improvement of current control strategies for this pest.

The co-evolutionary history of plant–insect interactions is estimated to be about 400 million years (Richards et al. 2016). Consequently, almost all aspects of herbivorous insects, including growth, population dynamics, and gene expression, etc., can be influenced by host plants (Qin et al. 2017). Generally, herbivorous insects have specific food utilization spectrums (Schoonhoven et al. 2005, Poelman et al. 2008, Wetzel et al. 2016). The occurrence, density and growth of herbivores are influenced by access to suitable plant food (Ibanez et al. 2013, Yang et al. 2013). And, this may also promote pest plague or outbreaks (Cease et al. 2012, Huang et al. 2017a). For example, the plague population of *Locusta migratoria manilensis* (Meyen) (Orthoptera: Oedipodidae) was found to have strongly correlated with the plant species, *Phragmites australis* (Cav.) Trin (Poales: Poaceae) (Ji et al. 2007). The desert locusts, *Schistocerca gregaria*, grew larger, developed faster, had higher survival, and reproduced more and earlier when reared on high-nitrogenous plant leaves. This may possibly contribute to the successful buildup of the desert locust populations, which subsequently could lead to upsurges and plagues (White 1976, van Huis et al. 2008). Also, the egg hatching rate, larval survival, and reproductive rates of *Spodoptera frugiperda* reared on corn were all higher than those reared on *Fagopyrum esculentum* (Moench.) (Polygonales: Polygonaceae), *Coix chinensis* (Tod.) (Poales: Poaceae), and *Phaseolus vulgaris* (Linn.) (Rosales: Leguminosae) (Li et al. 2019).

Growth and occurrence of herbivorous insects are significantly influenced by host plants, which is in turn underpinned by genetic mechanisms (Roy et al. 2016, Turlings and Erb 2018). Such genes in herbivorous insects may be associated with signaling, transporters, development, stress resistance, digestion, immunity, nutrition metabolites, or detoxification (Qin et al. 2017, Chang et al. 2019). For example, the grasshopper, *Oedaleus asiaticus* (Bey-Bienko) (Orthoptera: Oedipodidae) had a large variation in gene profiles when fed on unsuitable food plants. Genes related to DNA replication (endonuclease-reverse transcriptase, DNA primase large subunit, DNA polymerase alpha catalytic subunit, et al.), biosynthesis, and metabolism (6-phosphogluconate dehydrogenase, lipoyltransferase 1, putative fatty acyl-CoA reductase, peptidyl-prolyl isomerase-1, protein disulfide-isomerase, et al.) were downregulated significantly and some genes related to detoxification (cytochrome P450 6k1 and carboxylesterase) were upregulated significantly (Huang et al. 2017a,b). Studies on such change could enhance our understanding of the relationship between herbivorous insects and their host plants.

*Erythroneura sudra* (Hemiptera: Cicadellidae) is a serious sucking pest, especially for Rosaceae fruit tree species in China (Xin 2008). Attacked leaves can reduce tree photosynthesis and consequently decrease fruit production (Chen 2012, Fornasiero et al. 2016). So far, knowledge on the gene response of *E. sudra* to feeding on different host plants is currently limited. Therefore, it is required that more studies are conducted to provide detailed information on this relationship. We used RNA-Seq to explore the possible transcription mechanisms underlying responses in *E. sudra* when it fed on three different plant species. Our goal was to unravel how different host plants affected the regulation of gene expressions in *E. sudra.* Further, it was also to apply such knowledge for the development of improved management strategies to control this pest.

## Materials and Methods

### Field Survey

The study site was located in Yi county (43.862°N, 116.028°E), southern Shandong province, China. In this region, *E. sudra* is becoming a major leaf pest of fruit trees (Supp Fig. 1 [online only]). Three plots, respectively, planted by peach (*Amygdalus persica*), apple (*Malus domestica*), and cherry (*Cerasus pseudocerasus*) for 5 yr, were selected to survey the occurrence density of *E. sudra* in 2020. No insecticides were applied to the plots during the survey.

In total, 10 trees were randomly selected in each plot, to survey *E. sudra* occurrence and density. In each tree, we randomly selected 15 leaves from the top (5 leaves), middle (5 leaves), and bottom (5 leaves) to record the numbers of *E. sudra*. Then, we derived a relative *E. sudra* density for each tree (number of individuals per 15 leaves). The field survey was conducted from 6.00 a.m. to 8.00 a.m. when the *E. sudra* were less active (Xin 2008, Chen 2012). This survey was conducted two times per month from May to September.

### Feeding Trial

A 2-yr feeding trial were conducted during mid-July and late-August in 2019 and 2020 to determine how *C. pseudocerasus*, *M. domestica*, and *A. persica* affected the survival and development time of *E. sudra*. In each year, a total of 300 first-instar nymphs of *E. sudra* were starved for 24 h and assigned to 15 plastic insect-breeding cages. Then, all boxes were maintained under an 13:11 (L:D) h light regime at a RH of 70% and a temperature of 28°C.

To obtain fresh leaves to feed leafhoppers, we used gauze to protect leaves of the fruit tree species from March to August in each year. Fresh leaves (5 g) were harvested daily from each tree species and supplied as food to the leafhoppers. Each treatment was conducted five times. The leaves were replaced every 24 h and leafhoppers were inspected daily until all surviving individuals became adults. Developmental time (days) of leafhoppers was calculated using the same method described by Li et al. (1987). Survival rate (%) of leafhoppers was calculated by the number of surviving adults/the initial number of first-instar nymphs.

### Transcriptome Sequencing

We collected three samples of leafhoppers from each of three treatments in 2020. Each sample consisted of five *E. sudra* adults (one chosen randomly from each of the five replicates). In total, nine samples were prepared. RNA of these samples was extracted using TRIzol reagent (Invitrogen, Carlsbad, CA). Then, the NEBNext Ultra RNA Library Prep Kit for Illumina (New England Biolabs, Ipswich, USA) was used to generate the sequencing libraries. Finally, the Illumina HiSeqTM 4000 platform (Illumina Inc., San Diego, CA) was used to sequence the libraries.

To obtain high-quality clean reads, we removed the adaptor-containing sequences, poly-N, and low-quality reads. The remaining clean reads were further used in the assembly and gene abundance calculation. Trinity was used to carry out de novo assembly of the transcriptome (Grabherr et al. 2011). All nine transcriptomes were de novo assembled. Unigenes were used for BLASTX searches with an *E*-value < 10^−5^ in the following databases: Nt, KOG, Nr, Swiss-Prot, KO, PFAM, and Gene Ontology (GO).

### Differential Expression Analysis

SOAPaligner/soap2 was used to remap the sequenced reads for each sample to the assembled transcriptome (Li et al. 2009). Gene expression values were quantified as FPKM by RSEM (Li et al. 2011). Differentially expressed genes (DEGs) were detected using the DESeq2 package in R (Anders et al. 2010). Transcripts with a minimum two-fold difference (|log_2_·Fold_change| > 1) in expression and adjusted *P-*values < 0.05 were considered differentially expressed.

DEGs were annotated to the GO database using the GOseq R packages (Young et al. 2010), and mapped to pathways in the KEGG database using the KOBAS software according to *P*-value < 0.05 (Mao et al., 2005).

### Validation by Quantitative Real-Time PCR

In total, 12 candidate DEGs (Supp Table 1 [online only]) were chosen for validation by quantitative real-time PCR (qRT-PCR). The gene-specific primers are provided in Supp Table 2 (online only). We collected one adult randomly from each replicate cage, and extracted their total RNA and synthesized their cDNA using the AMV reverse transcriptase (Invitrogen). We conducted the qRT-PCR according to the following conditions: denaturation (95°C, 2 min; 40 cycles, 94°C, 10 s), annealing (59°C, 10 s), and extension (72°C, 40 s). β-Actin was used as the reference gene. Relative expression of each gene was analyzed by the 2^−ΔΔCT^ method. Three technical replicates were conducted for each gene.

### Data Analysis

Tukey’s HSD post hoc (one-way ANOVA, SAS version 8.0 software) was used to compare the relative density, developmental time, and survival rate of leafhopper after verifying normality. Holm–Sidak post hoc test was used to compare the relative gene expression levels between groups. *P* < 0.05 was considered statistically significant.

## Results

### Occurrence

**Fig. 1.**
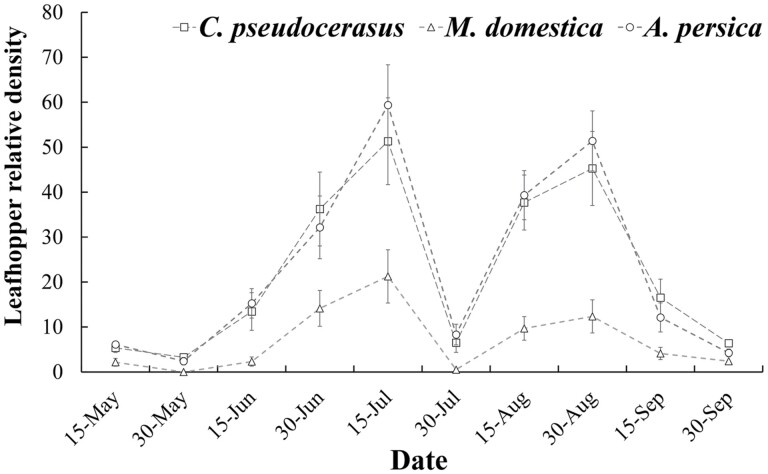
The occurrence and density of *E. sudra* (mean number of individuals per 15 leaves ± SD) on the three tree species from May to September in 2020.

The field survey from May to September in 2020 (Fig. 1) showed that the occurrence density of *E. sudra* on the tree species had two peaks. The first peak occurred on 15 July, with recorded relative densities on *A. persica*, *C. pseudocerasus*, and *M. domestica* as 59.37 ± 9.66, 51.32 ± 8.96, and 21.26 ± 5.92, respectively. The second peak occurred on 30 August, with recorded relative densities on *A. persica*, *C. pseudocerasus*, and *M. domestica* as 45.27 ± 6.69, 51.39 ± 8.25, and 12.36 ± 3.68, respectively. The relative densities of *E. sudra* on *M. domestica* were significantly lower at the two peaks, compared to the other two fruit trees (the first peak: *F* = 10.68, df = 2, 447, *P* < 0.001; the second peak: *F* = 13.32, df = 2, 447, *P* < 0.001).

### Growth Performance

**Fig. 2.**
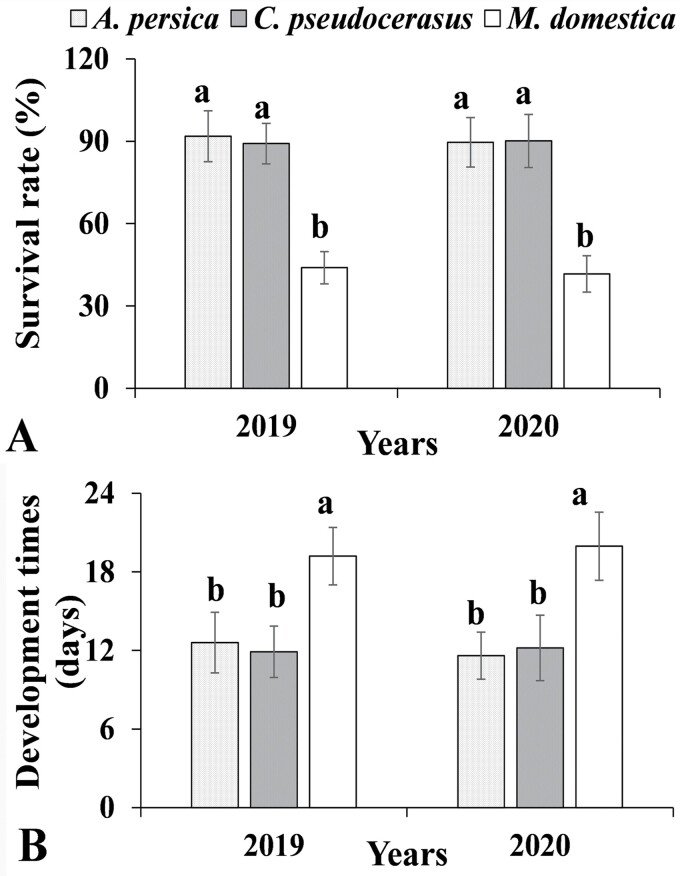
The survival rate (% ± SD) (A) and developmental time (mean days ± SD) (B) of *E. sudra* from first instar to adults when reared on *A. persica*, *C. pseudocerasus*, and *M. domestica*. Significant differences between the leafhoppers feeding on three plant species within the same year are indicated by lowercase letters (ANOVA, Tukey’s HSD analysis, *P* < 0.05).

The 2-yr feeding trial showed that *E. sudra* had significant higher survival rates (2019: *F* = 5.65; df = 2, 12; *P* = 0.003; 2020: *F* = 7.91; df = 2, 12; *P* < 0.001) and faster developmental times (2019: *F* = 2.68; df = 2, 12; *P* = 0.035; 2020: *F* = 3.96; df = 2, 12; *P* = 0.021) on *A. persica* and *C. pseudocerasus* than on *M. domestica* (Fig. 2).

### Transcriptome Analysis

Transcriptomes (accession number SRR14224867) of *E. sudra* when it was fed on the three tree species generated 40,411 unigenes (Table 1; Supp Table 3 [online only]). Of these, 17,585 (43.51%) were successfully annotated by NCBI Nr, 13,534 (33.49%) by Swiss-Prot, 14,028 (34.71%) by GO, 14,030 (34.71%) by PFAM, 20,989 (51.94%) by KO, 8,141 (20.15%) by KOG, and 6,538 (16.17%) by Nt (Supp Table 4 [online only]).

**Table 1.** Statistics for the assembled sequences

Group name	Number
Total assembled bases	186,785,416
Total number of unigenes	40,411
GC percentage (%)	46.18
Unigene N50 (bp)	1,466
Unigene N90 (bp)	438
Maximum unigene length (bp)	27,603
Minimum unigene length (bp)	301
Average unigene length (bp)	1,100

### DEGs in *E. sudra*

**Fig. 3.**
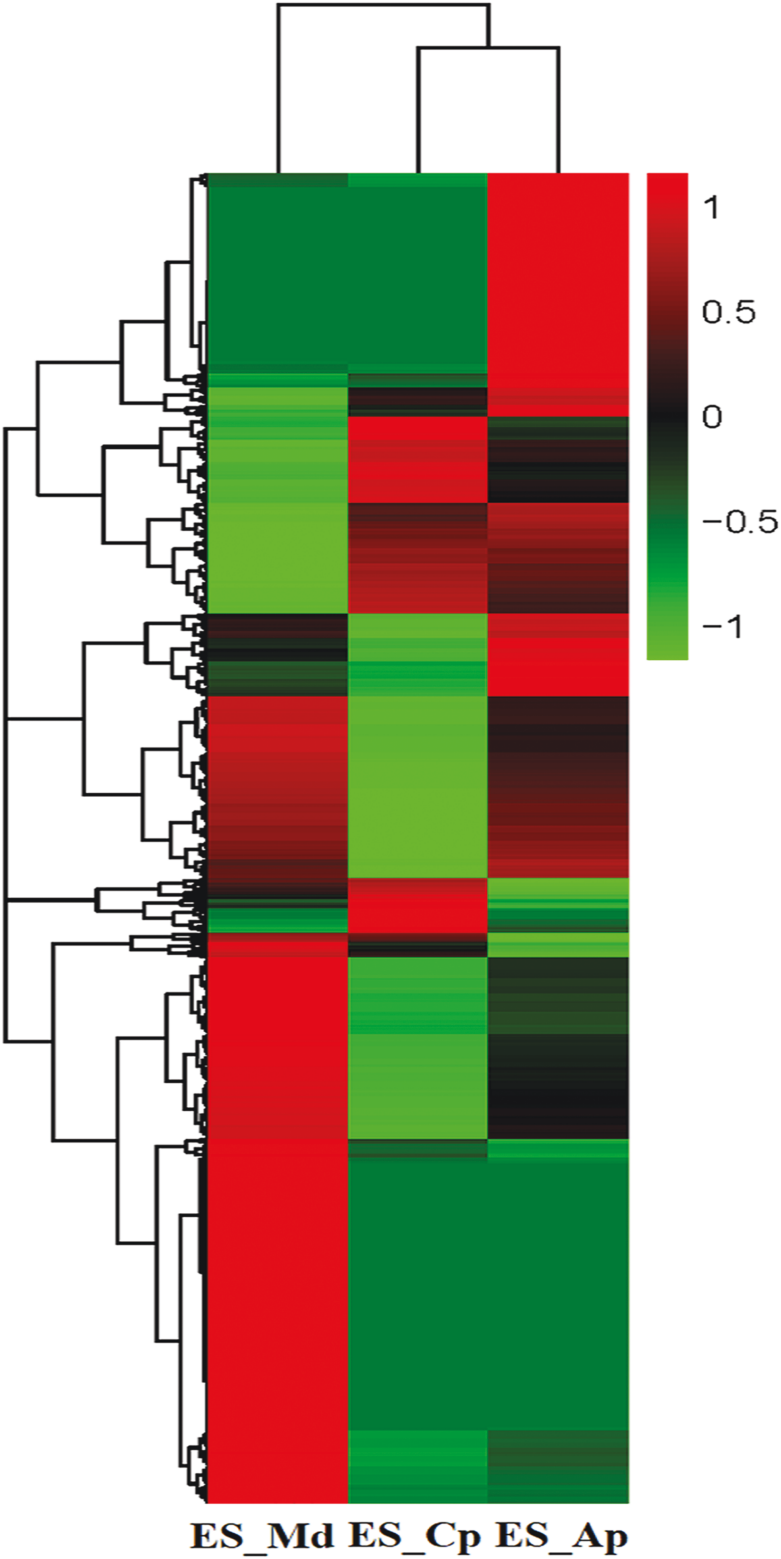
Cluster analysis of DEGs in *E. sudra* when it fed on three different plant species. Genes in red show upregulation, and those in green show downregulation. A change from red to green indicates a decrease in value of normalized expression levels (*z*-scores). ES_Md (*E. sudra* feeding on *M. domestica*), ES_Cp (*E. sudra* feeding on *C. pseudocerasus*), and ES_Ap (*E. sudra* feeding on *A. persica*).

**Fig. 4.**
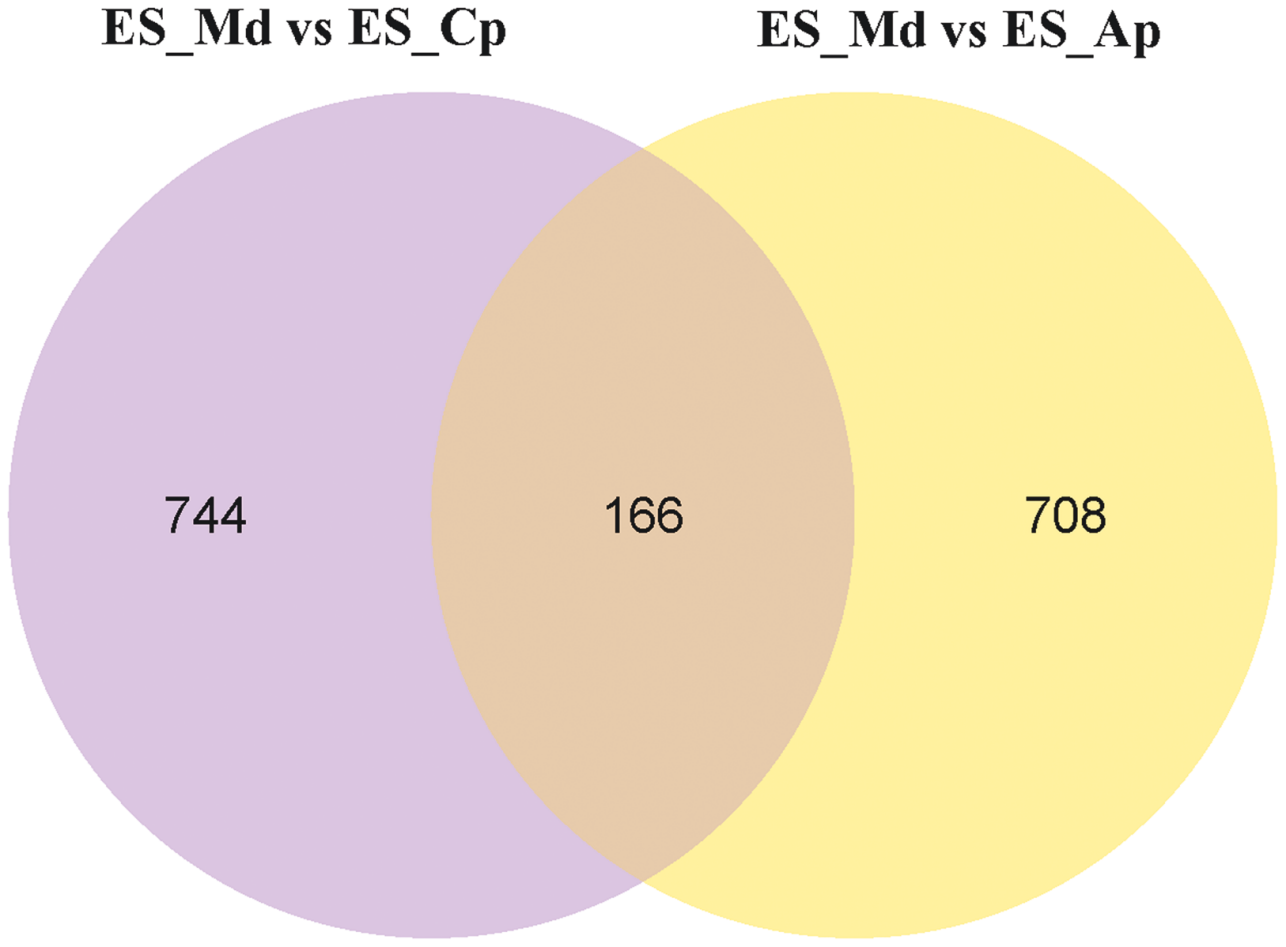
Venn diagram showing the DEGs that were similar between *E. sudra* individuals that fed on *M. domestica* and those that fed on *C. pseudocerasus* or *A. persica*. ES_Md (*E. sudra* feeding on *M. domestica*), ES_Cp (*E. sudra* feeding on *C. pseudocerasus*), and ES_Ap (*E. sudra* feeding on *A. persica*).

DEGs were generated by comparing ES-Md (sample feeding on *M. domestica*) versus ES-Ap (sample feeding on *A. persica*) (677 downregulated, 197 upregulated), ES-Md versus ES-Cp (sample feeding on *C. pseudocerasus*) (645 downregulated, 265 upregulated), and ES-Cp versus ES-Ap (66 downregulated, 108 upregulated) (Table 2). *Erythroneura sudra*’s feeding on *M. domestica* generated the highest number of upregulated or downregulated genes. The cluster analysis of DEGs (Fig. 3) also showed a significant genetic variation in *E. sudra*’s feeding on *M. domestica*. In total, 166 genes (77 downregulated, 89 upregulated) were differentially expressed when *E. sudra* fed on *M. domestica*, compared separately to when it fed on the other two plant species (Fig. 4; Supp Tables 5 and 6 [online only]).

**Table 2.** *Erythroneura sudra* DEGs following feeding on the three host plant species

Leafhopper comparison	Downregulated genes	Upregulated genes
ES-Md vs ES-Ap	677	197
ES-Md vs ES-Cp	645	265
ES-Cp vs ES-Ap	66	108

ES_Md (*E. sudra* feeding on *M. domestica*), ES_Cp (*E. sudra* feeding on *C. pseudocerasus*), and ES_Ap (*E. sudra* feeding on *A. persica*), respectively.

The DEGs between when *E. sudra* fed on *M. domestica* and when it fed on the other two trees were assigned to 10 GO terms using the GOseq packages in R (corrected *P*-value < 0.05) (Table 3). Downregulated GO terms included ‘structural constituent of cuticle’, ‘carbohydrate metabolic process’, ‘chitin binding’, ‘starch metabolic process’, and ‘biosynthetic process’. Upregulated GO terms included ‘antioxidant activity’, ‘regulation of apoptotic process’, ‘response to oxidative stress’, ‘response to reactive oxygen species’, and ‘xenobiotic metabolic process’.

**Table 3.** GO enrichment analysis (corrected *P*-value < 0.05) of the DEGs of *E. sudra* that fed on *M. domestica* compared to those that fed on *C. pseudocerasus* or *A. persica*

GO terms	Upregulation/downregulation	Number of genes for ES_Md vs ES_Ap	Number of genes for ES_Md vs ES_Cp
Structural constituent of cuticle	Down	16	14
Carbohydrate metabolic process	Down	—	13
Chitin binding	Down	8	10
Starch metabolic process	Down	15	11
Biosynthetic process	Down	13	—
Antioxidant activity	Up	13	12
Regulation of apoptotic process	Up	10	—
Response to oxidative stress	Up	—	12
Response to reactive oxygen species	Up	12	—
Xenobiotic metabolic process	Up	11	9

ES_Md (*E. sudra* feeding on *M. domestica*), ES_Cp (*E. sudra* feeding on *C. pseudocerasus*), and ES_Ap (*E. sudra* feeding on *A. persica*), respectively. ‘—’ indicates the corrected *P*-value > 0.05 and therefore not significantly different.

The DEGs between when *E. sudra* fed on *M. domestica* and when it fed on the other two trees were assigned to 11 pathways using the KOBAS software (*q*-value < 0.05) (Table 4). The downregulated pathways included ‘carbohydrate digestion and absorption’, ‘insulin signaling pathway’, ‘glycolysis/luconeogenesis’, ‘fatty acid metabolism’, ‘ribosome’, ‘N-glycan biosynthesis and cutin’, ‘suberine and wax biosynthesis’. The upregulated pathways included ‘peroxisome’, ‘metabolism of xenobiotics by cytochrome P450’, ‘FoxO signaling pathway’, and ‘apoptosis’.

**Table 4.** KEGG enrichment analysis (*q*-value < 0.05) of the DEGs in *E. sudra* that fed on *M. domestica* compared to those that fed on *C. pseudocerasus* or *A. persica*

Pathway	Upregulation/downregulation	Number of genes for ES_Md vs ES_Ap	Number of genes for ES_Md vs ES_Cp
Carbohydrate digestion and absorption	Down	8	12
Insulin signaling pathway	Down	13	9
Glycolysis/gluconeogenesis	Down	10	—
Fatty acid metabolism	Down	13	7
Ribosome	Down	17	15
N-glycan biosynthesis	Down	11	—
Cutin, suberine and wax biosynthesis	Down	9	6
Peroxisome	Up	7	—
Metabolism of xenobiotics by cytochrome P450	Up	9	8
FoxO signaling pathway	Up	6	—
Apoptosis	Up	—	6

ES_Md (*E. sudra* feeding on *M. domestica*), ES_Cp (*E. sudra* feeding on *C. pseudocerasus*), and ES_Ap (*E. sudra* feeding on *A. persic*). ‘—’ indicates a corrected *P*-value > 0.05 and therefore not significantly different.

### Gene Expression by qRT-PCR

**Fig. 5.**
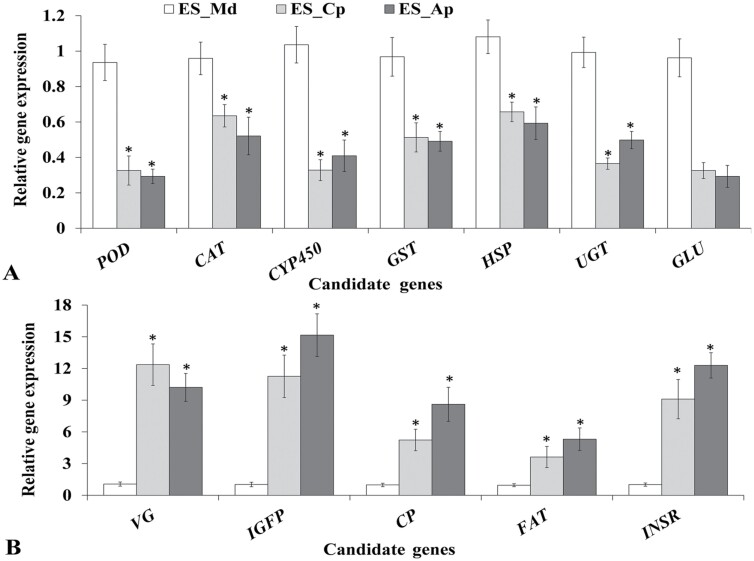
qRT-PCR analysis of 12 candidate genes. (A) Significantly up-regulated candidate genes in *E. sudra* fed on *M. domestica*, (B) Significantly down-regulated candidate genes in *E. sudra* fed on *M. domestica*. The results were evaluated using the 2^−ΔΔCT^ method. The 2^−ΔΔCT^ values of controls were set to one to calibrate the relative gene expression levels. ES_Md (*E. sudra* feeding on *M. domestica*), ES_Cp (*E. sudra* feeding on *C. pseudocerasus*), and ES_Ap (*E. sudra* feeding on *A. persic*). Bars represent mean ± SD values. * notes *P* < 0.05 versus ES_Md.

The stress-resistant or detoxification enzyme-related *POD*, *CAT*, *CYP450*, *GST*, *HSP*, *UGT*, and *GLU* were all upregulated in *E. sudra* when it fed on *M. domestica* (Fig. 5A). The cuticle biosynthesis and development regulation-related *VG*, *IGFP*, *CP*, *FAT*, and *INSR* were downregulated (Fig. 5B). The qRT-PCR results and the RNA-Seq data showed significant correlations (*r*^2^ = 0.9783, *P* < 0.05).

## Discussion

The occurrence and growth of herbivorous insects are closely associated with their host plants (Agrawal et al. 2015, Wetzel et al. 2016). In this study, we also found that the biological adaptability of *E. sudra* to their host plant is variable. *Erythroneura sudra* had higher population densities and superior growths when it fed on *C. pseudocerasus* and *A. persica*, which suggested that these tree species were more preferred by *E. sudra* than *M. domestica*. This implies that mass plantings of *C. pseudocerasus* and *A. persica* in the study area may promote a high occurrence of *E. sudra* population or even outbreaks. Hence, farmers should strengthen the management of *E. sudra* populations to avoid population explosions in this area. However, the reduced growth performance and lower density of *E. sudra* recorded on *M. domestica* compared to *C. pseudocerasus* and *A. persica* does not necessarily qualify it as undesirable, as it nevertheless supported a lower population of *E. sudra* that fed on it.

Variations in insect growth and occurrence on different host plants are underpinned by gene regulation (Turlings and Erb 2018). In this study, we also found that *E. sudra* had specific genetic adaptability to different host plants. *Erythroneura sudra* had significant variation in gene expressions when fed on *M. domestica*. Not surprisingly, little variation was observed in gene expression of leafhoppers that fed on *A. persica* and *C. pseudocerasus*, because their growth performance and density were not significantly different.

*Erythroneura sudra* feeding on *M. domestica* had many up-regulated genes related to ‘response to oxidative stress’, ‘stress-resistance’, and ‘xenobiotic metabolic process’. This was probably in response to stress from feeding on *M. domestica*. For example, the candidate gene for qRT-PCR, *HSP* (heat shock proteins), *POD* (peroxiredoxin), and *CAT* (catalase) are widely known to be highly induced when animals are confronted with environmental stress (Despres et al. 2007, Powell et al. 2011, Kumar et al. 2016, Li et al. 2016, Nojima et al. 2019). The upregulation of stress-resistance genes from the feeding on *M. domestica* corresponded to a significant lower growth and density of *E. sudra*. Generally, upregulation of these genes ameliorates stress in organisms (King and Macrae 2015). The *CYP450* (cytochrome P450s), *GST* (glutathione-*S*-transferases), *UGT* (UDP-glycosyltransferases), and *GLU* (membrane beta-glucosidase) in herbivorous insects can modify the host plant’s toxic compounds, which are ingested, to render them less toxic (Zhang et al. 2012, Schuler and Berenbaum 2013, Xu et al. 2015, Poreddy et al. 2015, Pan et al. 2020). Undoubtedly, *E. sudra* has evolved complex strategies, such as the induction of many stress resistance-related genes to overcome the adverse effects from diet stress. Also, these genes were mainly enriched in the pathways of metabolism of xenobiotics by cytochrome P450, apoptosis, peroxisome, and FoxO signaling pathway, which have been confirmed to be related to stress-resistance regulation (Aparna et al. 2004, Lehtinen et al. 2006, Despres et al. 2007, Davies et al. 2013). Upregulation of such genes or pathways is beneficial for leafhoppers to overcome the potential diet stress for survival.

In addition, we found that leafhoppers that fed on *M. domestica* had many downregulated genes related to ‘structural constituent of cuticle’, ‘carbohydrate metabolic process’, ‘biosynthetic process’, and ‘development regulation’. For example, the candidate gene for qRT-PCR, *VG* (vitellogenin), *IGFP* (insulin-like growth factor-binding protein), *CP* (cuticle protein), *FAT* (fatty acid synthase), and *INSR* (insulin receptor) have been identified to be involved in insect development (Taguchi and White 2008, Charles 2010, Badisco et al. 2013, Hu et al. 2019). The downregulation of these genes and their enriched pathways (e.g., insulin signaling pathway, carbohydrate digestion and absorption) also may be responses to toxic substances or nutritional deficits in such plants and that can reduce insect development or reproduction. These genes and pathways may have played a role in the reduced growth performance of *E. sudra*. Such responses to *M. domestica* may also be vital for leafhopper survival. Future studies should identify the role of these significantly changed genes in leafhopper feeding on different plant foods.

The biological and genetic adaptations, including growth, density, and gene expressions of the leafhopper *E. sudra* to different host plants, are variable. The underlying factors may be related to plant defense responses or nutritive compounds (Richards et al. 2016, Züst and Agrawal 2017). For example, *A. persica* and *C. pseudocerasus* may have important nutrients, such as fatty acids or vitamins, or have appropriate C/N ratio (Ibanez et al. 2013), that are vital for leafhopper growth. *Malus domestica*, on the other hand, may have nutritional deficits or possess some special substances that induced stress in the leafhoppers. Future research should focus on the potential effects of such chemicals on gene expressions in the leafhopper. Undoubtedly, such information could enhance our understanding of the relationship between herbivorous insects and their host plants. Additionally, it could contribute to improvements in current control and management strategies for this pest.

We demonstrated by transcriptome analysis that *E. sudra* had a lower density and decreased growth when it fed on leaves of *M. domestica* than on *A. persica* or *C. pseudocerasus*. Here, we only studied the same 166 DEGs between *E. sudra* that had fed on *M. domestica* compared to that which fed on the other two tree species. We also found other DEGs between *E. sudra* when it fed on *A. persica* and *C. pseudocerasus*. Determination of the selective factors that influenced these different expression patterns requires future study.

## Supplementary Material

ieac037_suppl_Supplementary_Material
