# Supplementary material for: Occurrence, Density, and Transcriptomic Response of the Leafhopper Erythroneura sudra (Hemiptera: Cicadellidae) When Confronted With Different Fruit Tree Species
Source: J Insect Sci. 2022 Jun 28;22(3):18. doi: 10.1093/jisesa/ieac037 (PMC9239223; doi:10.1093/jisesa/ieac037)
Supplement: ieac037_suppl_Supplementary_Material [file ieac037_suppl_supplementary_material.pdf]

Occurrence, density and transcriptomic response of the leafhopper *Erythroneura sudra* (Hemiptera: Cicadellidae) when fed on leaves of different fruit trees

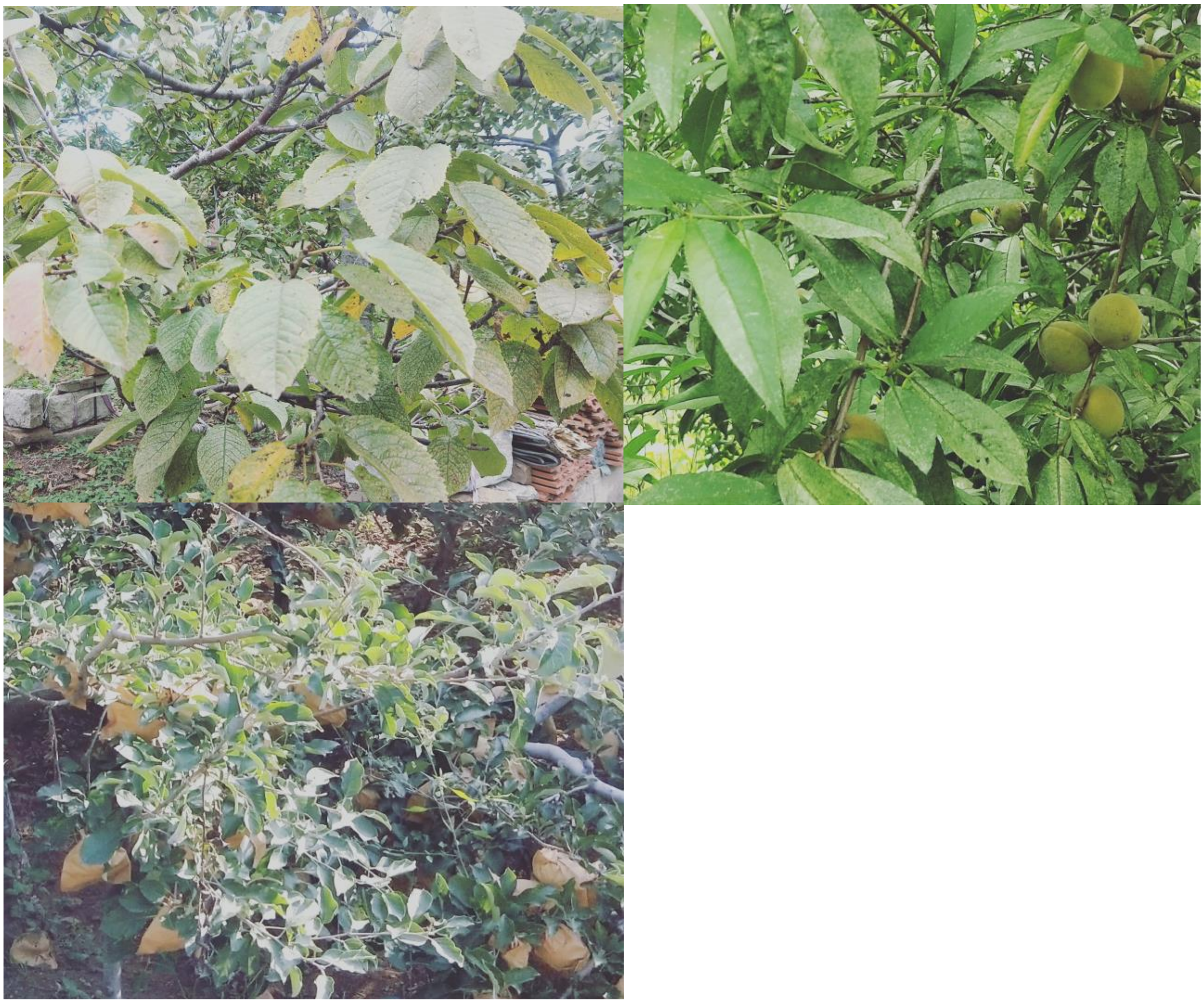

Figure S1 The damage of *E. sudra* to cherry (*Cerasus pseudocerasus*), peach (*Amygdalus persica*) and apple (*Malus domestica*)

Table S1 The selected 12 DEGs for qRT-PCR analysis

Gene ID	Description	Abbreviation	log2.Fold_change ES_Md VS. ES_Ap	log2.Fold_change ES_Md VS. ES_Cp
Cluster-19019.2132	peroxiredoxin [Nilaparvata lugens]	*POD*	10.16927685	6.174359249
Cluster-1547.0	catalase [Harpegnathos saltator]	*CAT*	8.829506781	3.066932372
Cluster-5177.0	cytochrome P450 6a13 [Cryptotermes secundus]	*CYP450*	3.749362084	4.088665445
Cluster-19019.3758	glutathione S-transferase [Apolygus lucorum]	*GST*	3.896363427	2.680641499
Cluster-27.0	heat shock protein 70 [Leishmania braziliensis]	*HSP*	7.881373167	5.624837707
Cluster-19019.13282	UDP-glucuronosyltransferase 2B33 [Nilaparvata lugens]	*UGT*	2.548765702	4.140533965
Cluster-2308.0	membrane beta-glucosidase 1 [Physarum polycephalum]	*GLU*	11.66324693	12.77840303
Cluster-5815.0	vitellogenin [Homalodisca vitripennis]	*VG*	-6.430761322	-2.971863792
Cluster-8830.0	insulin-like growth factor-binding protein-related protein 1 [Cryptotermes secundus]	*IGFP*	-3.358876859	-5.821629852
Cluster-19019.10911	cuticular protein [Nilaparvata lugens]	*CP*	-8.029723962	-9.039122327
Cluster-5034.0	fatty acid synthase [Rhopalosiphum maidis]	*FAT*	-3.901906417	-7.35905604
Cluster-11742.0	insulin receptor 2 [Oncopeltus fasciatus]	*INSR*	-2.95732896	-3.03440695

Table S2 Designed sequences of qRT-PCR primers for 12 genes

Gene description	Sequence of primers (5' to 3')	
peroxiredoxin *POD*	Forward	CTAGAGACCTGAATAGGAAT
	Reverse	ATCAGTCTTATACTTGACCA
catalase *CAT*	Forward	GAGTCGGGATTGCAGCACG
	Reverse	ACTTGGGAAAGTCGCCCTT
probable cytochrome P450 6a13 *CYP450*	Forward	TCCTCCAACTGGTTTTGGCAC
	Reverse	CCAGATATGGGAATGAATACT
glutathione S-transferase *GST*	Forward	TCTTAACAGGTCCTTTCCAATC
	Reverse	TTGGTTAGTTTTCATGAACTTG
heat shock protein 70 *HSP*	Forward	TTCGACATCGACGCGAACG
	Reverse	CGGGCTGGAGAACTACGCGT
UDP-glucuronosyltransferase 2B33 *UGT*	Forward	CTAGTACGGTTTATAATGACTC
	Reverse	TGCCATATATTTGTAAGATTG
membrane beta-glucosidase 1 *GLU*	Forward	AGCTCTTAGCCCTGTTGCTGGA
	Reverse	CGCTAACGCCGTGAAGGCTAA
vitellogenin *VG*	Forward	CGAGACTGGCTGGATTTCTCTCC
	Reverse	CTTGCTCTGACCTTGGAACTCG
insulin-like growth factor-binding protein-related protein 1 *IGFP*	Forward	GCTACAACGCCTCCATCTTCAAG
	Reverse	AGGCCCTGAGACTACGAACGCC
fatty acid synthase *FAT*	Forward	ATAATGGTGGGTGAGAGCAGG
	Reverse	GAGCCGCTGGTGCGTATCCAG
cuticle protein *CP*	Forward	CAGCTACATTCCTGGTTACCTAT
	Reverse	CTGGAACCAGATGGAATGGTTG
insulin receptor 2 *INSR*	Forward	CAGGAATTATGGGATTGGGACT
	Reverse	CTTGAAATCGCTACAGATCTTAA
β-actin	Forward	CAGCTGTATCCTGCATACCGC
	Reverse	CGTCGATGTACGACAGCTGTT

Table S3 Summary of RNA-seq metrics from *Erythroneura sudra* transcriptomes.

Key: ES_Md (*E. sudra* feeding on *M. domestica*), ES_Cp (*E. sudra* feeding on *C. pseudocerasus*) and ES_Ap (*E. sudra* feeding on *A. persic*), respectively.

**sample**	**raw_reads**	**clean_reads**	**error_rate**	**Q20**	**Q30**	**GC_pct**
ES_Md 1	21568189	20671562	0.03	97.76	93.44	42.36
ES_Md 2	20968256	20659781	0.03	97.68	93.26	47.68
ES_Md 3	21365973	20898421	0.03	97.52	93.52	46.82
ES_Cp 1	22146135	20981931	0.03	97.70	93.36	42.65
ES_Cp 2	21985361	20996521	0.03	97.26	92.69	46.86
ES_Cp 3	20965963	20562132	0.03	97.82	92.98	48.01
ES_Ap 1	21801530	20583601	0.03	97.76	93.62	48.11
ES_Ap 2	21632108	20468211	0.03	97.50	93.12	46.21
ES_Ap 3	21203068	20963256	0.03	97.62	93.25	45.62

Table S4 Annotation results of unigenes

Database	Number.of.Unigenes	Percentage
Annotated in NR	17585	43.51
Annotated in NT	6538	16.17
Annotated in KO	20989	51.94
Annotated in KOG	8141	20.15
Annotated in SwissProt	13534	33.49
Annotated in PFAM	14030	34.71
Annotated in GO	14028	34.71
Annotated in all Databases	3403	8.42
Total Unigenes	40411	100

Table S5 The same differentially expressed genes (qvalue <0.05, |log2.Fold_change|>1, only annotated and down-regulated genes) of *E. sudra* fed on *M. domestica* compared with individuals feeding the other two plants *C. pseudocerasus*, *A. persic*.

Gene id	Nr annotation	log2.Fold_change ES_Md vs ES_Ap	log2.Fold_change ES_Md vs ES_Cp
Cluster-19019.7524	cuticle protein 7-like [Cimex lectularius]	-9.207304872	-14.7869239
Cluster-19019.10911	cuticular protein [Nilaparvata lugens]	-9.039122327	-8.029723962
Cluster-19019.1088	cuticle protein 19-like [Leptinotarsa decemlineata]	-13.00407801	-8.256337712
Cluster-19019.1080	Insect cuticle protein, partial [Oryctes borbonicus]	-6.876359771	-7.867759962
Cluster-19019.124	cuticle protein 8 [Halyomorpha halys]	-12.77403181	-7.895956862
Cluster-8199.0	endocuticle structural glycoprotein SgAbd-2-like [Cimex lectularius]	-8.490498439	-8.4576289
Cluster-19019.7069	pupal cuticle protein 36a-like isoform X2 [Nilaparvata lugens]	-9.657263087	-13.81986093
Cluster-16505.0	cAMP-dependent protein kinase catalytic subunit [Cimex lectularius]	-6.91101059	-9.233086624
Cluster-19019.10689	cuticle protein 8-like [Halyomorpha halys]	-7.703817906	-8.901033871
Cluster-21105.0	PREDICTED: larval cuticle protein A2B-like [Drosophila rhopaloa]	-7.406510288	-13.21349797
Cluster-21718.0	PREDICTED: cuticle protein 8 [Rhagoletis zephyria]	-6.158906257	-13.21426632
Cluster-19019.6811	Endocuticle structural glycoprotein SgAbd-2, partial [Blattella germanica]	-6.301552763	-7.273641586
Cluster-19019.6135	cuticle protein 21-like isoform X2 [Halyomorpha halys]	-6.02856736	-8.601303289
Cluster-19019.349	cuticular protein [Nilaparvata lugens]	-6.107841346	-7.850992881
Cluster-6342.0	cuticle protein 19-like [Cimex lectularius]	-7.966002389	-12.89716896
Cluster-17402.0	pro-resilin [Lucilia cuprina]	-11.96641005	-6.969471357
Cluster-21009.0	flexible cuticle protein 12-like [Ctenocephalides felis]	-6.783821316	-12.88408533
Cluster-7815.0	cuticle protein-like [Cimex lectularius]	-11.85611529	-7.303169371
Cluster-11731.0	putative galactose-1-phosphate uridylyltransferase [Blattella germanica]	-6.55536209	-9.86862069
Cluster-15798.0	PREDICTED: probable chitinase 3 [Dendroctonus ponderosae]	-6.263944763	-7.405323159
Cluster-787.0	glycoside hydrolase family 6 protein [Stylonychia lemnae]	-8.879563505	-8.818660338
Cluster-11316.0	Dynein heavy chain 7 [Blattella germanica]	-11.81411926	-12.6202181
Cluster-8532.0	glycerol kinase-like isoform X1 [Zootermopsis nevadensis]	-5.862195169	-7.569222978
Cluster-21161.0	glucose-6-phosphate 1-epimerase [Zootermopsis nevadensis]	-7.02441538	-12.58587283
Cluster-19019.503	Phosphoacetylglucosamine mutase-like Protein [Tribolium castaneum]	-7.356538744	-12.52987658
Cluster-2624.0	LOW QUALITY PROTEIN: probable glycosidase crf2 [Nilaparvata lugens]	-5.633008797	-6.18165118
Cluster-19019.5086	beta-galactosidase isoform X2 [Zootermopsis nevadensis]	-11.69812901	-9.530607499
Cluster-2598.0	GATA zinc finger domain-containing protein 14-like [Cephus cinctus]	-5.413816705	-8.617244058
Cluster-23378.1	GDP-fucose protein O-fucosyltransferase 1 [Frankliniella occidentalis]	-5.402472291	-6.363607075

Cluster-19019.1249	hypothetical protein LSTR_LSTR006211 [Laodelphax striatellus]	-7.218616529	-6.783826032
Cluster-18168.0	hypothetical protein LSTR_LSTR002461 [Laodelphax striatellus]	-5.770030839	-7.399017587
Cluster-8511.0	PREDICTED: peritrophin-48-like [Rhagoletis zephyria]	-5.926605053	-6.376440213
Cluster-19019.9759	vacuolar ATP synthase subunit ac39 [Liposcelis bostrychophila]	-11.43375988	-6.54018424
Cluster-19019.9757	40S ribosomal protein S14a [Nilaparvata lugens]	-11.43375988	-5.985910575
Cluster-415.0	60S ribosomal protein L32-like [Crassostrea virginica]	-11.4079293	-6.943553079
Cluster-8218.0	Ribosome biogenesis protein BRX1 [Blattella germanica]	-7.285199833	-8.393648177
Cluster-811.0	60S ribosomal protein L23a, putative [Bodo saltans]	-5.665752583	-6.116172855
Cluster-19267.0	poly [ADP-ribose] polymerase 12-like [Cryptotermes secundus]	-7.226620681	-7.09626928
Cluster-1422.0	60S ribosomal protein L15-A-like [Mizuhopecten yessoensis]	-8.358635331	-6.207725729
Cluster-19019.781	PREDICTED: juvenile hormone acid O-methyltransferase-like [Bemisia tabaci]	-9.012435047	-6.499551571
Cluster-7062.0	vitellogenin-like [Zootermopsis nevadensis]	-4.706855844	-12.13205038
Cluster-5815.0	vitellogenin [Homalodisca vitripennis]	-6.430761322	-2.971863792
Cluster-19019.13409	vitellogenin receptor [Nilaparvata lugens]	-6.102366072	-12.04908005
Cluster-12517.0	insulin-like peptide receptor [Cryptotermes secundus]	-8.989628707	-7.569194062
Cluster-11742.0	insulin receptor 2 [Oncopeltus fasciatus]	-2.95732896	-3.03440695
Cluster-12501.0	insulin-like growth factor-binding protein 7 [Cimex lectularius]	-8.213396491	-12.0322226
Cluster-17047.1	insulin-like receptor isoform X3 [Zootermopsis nevadensis]	-5.605396491	-12.02253033
Cluster-8830.0	insulin-like growth factor-binding protein-related protein 1 [Cryptotermes secundus]	-3.358876859	-5.821629852
Cluster-1679.0	glycogen(starch) synthase [Fonticula alba]	-3.007283626	-7.796916519
Cluster-19019.11724	cuticle protein 21-like [Nilaparvata lugens]	-3.028281197	-7.049894818
Cluster-19019.11759	AGAP010900-PA-like protein [Anopheles sinensis]	-3.171591785	-11.85931705
Cluster-19019.3215	putative ATP-dependent RNA helicase spindle-E [Cryptotermes secundus]	-4.47367783	-8.043445515
Cluster-3098.0	28S ribosomal protein S17, mitochondrial [Ostrinia furnacalis]	-4.211977951	-7.019384318
Cluster-5034.0	fatty acid synthase-like, partial [Rhopalosiphum maidis]	-3.901906417	-7.35905604
Cluster-787.0	glycoside hydrolase family 6 protein [Stylonychia lemnae]	-3.129660624	-8.927810436
Cluster-2136.0	carboxypeptidase B [Penaeus vannamei]	-6.040933562	-11.75535414
Cluster-19019.703	sugar transporter [Nilaparvata lugens]	-3.027647674	-6.91536344
Cluster-3948.0	phosphatidylinositol 4-kinase beta [Apis cerana]	-3.813574655	-11.72285104
Cluster-17163.2	dehydrogenase/reductase SDR family member 12-like isoform X1 [Nilaparvata lugens]	-8.917987555	-11.60068284
Cluster-1306.0	hypothetical protein EgrG_000335800 [Echinococcus granulosus]	-2.959319799	-5.365395161
Cluster-10935.0	PREDICTED: translation elongation factor 2 [Polistes canadensis]	-8.893376924	-11.56028177
Cluster-1823.0	hypothetical protein ACA1_144040 [Acanthamoeba castellanii str. Neff]	-6.040933562	-11.52292745

Cluster-19019.7832	PREDICTED: cytochrome P450 4c3 [Musca domestica]	-3.027647674	-8.628693986
Cluster-22786.0	uncharacterized protein LOC113209437 isoform X1 [Frankliniella occidentalis]	-3.813574655	-11.51588849
Cluster-16416.0	hypothetical protein LSTR_LSTR003779 [Laodelphax striatellus]	-8.917987555	-6.544787702
Cluster-373.0	glucose-regulated protein 78, putative [Trypanosoma vivax Y486]	-2.959319799	-8.538783653
Cluster-14229.0	PREDICTED: SUN domain-containing protein 1 [Microplitis demolitor]	-5.445188965	-7.243856838
Cluster-18462.0	uncharacterized protein LOC105275530 isoform X1 [Ooceraea biroi]	-4.732813197	-6.208409825
Cluster-19019.3721	PREDICTED: RNA-directed DNA polymerase from mobile element jockey-like [Amyelois transitella]	-3.344434179	-11.33680085
Cluster-17929.0	uncharacterized protein LOC114334883 [Diabrotica virgifera virgifera]	-2.735975385	-11.32010523
Cluster-19019.8783	dnaJ homolog subfamily C member 5 isoform X2 [Nilaparvata lugens]	-2.775129472	-5.075980305
Cluster-316.0	NADH dehydrogenase subunit 5 (mitochondrion) [Balanoglossus carnosus]	-8.284919349	-5.64261407
Cluster-19019.13374	hypothetical protein LSTR_LSTR004195 [Laodelphax striatellus]	-5.445188965	-11.31039389
Cluster-19019.13375	dopamine N-acetyltransferase-like isoform X2 [Cimex lectularius]	-4.732813197	-5.621054632
Cluster-16762.0	hypothetical protein LSTR_LSTR005445 [Laodelphax striatellus]	-3.344434179	-11.27313285

Table S6 The same differentially expressed genes (qvalue <0.05, |log2.Fold_change|>1, only annotated and up-regulated genes) of *E. sudra* fed on *M. domestica* compared with individuals feeding the other two plants *C. pseudocerasus*, *A. persic*.

Gene id	Nr annotation	log2.Fold_change ES_Md vs ES_Ap	log2.Fold_change ES_Md vs ES_Cp
Cluster-18290.0	PREDICTED: peroxiredoxin-5, mitochondrial [Fopius arisanus]	11.55754442	7.603747521
Cluster-19019.4963	glutaredoxin 3 [Cryptotermes secundus]	4.449067444	7.313253023
Cluster-19019.2132	peroxiredoxin [Nilaparvata lugens]	10.16927685	6.174359249
Cluster-19019.371	probable phospholipid hydroperoxide glutathione peroxidase isoform X1 [Melanaphis sacchari]	10.08861769	10.10713989
Cluster-19019.7882	peroxiredoxin-6 [Nilaparvata lugens]	4.527991399	10.0264839
Cluster-19019.5508	PREDICTED: peroxiredoxin-5, mitochondrial isoform X1 [Bemisia tabaci]	3.680394944	4.899481603
Cluster-19019.8215	Protein disulfide-isomerase [Cryptotermes secundus]	6.71363942	9.85023726
Cluster-19019.12778	PREDICTED: thioredoxin-like protein 1 [Nasonia vitripennis]	3.827921461	3.728059891
Cluster-864.0	PREDICTED: protein disulfide-isomerase A3-like [Saccoglossus kowalevskii]	3.631984817	3.555454913
Cluster-18009.0	myelin transcription factor 1 isoform X3 [Bombyx mori]	3.789867435	4.11002308
Cluster-17810.0	tRNA (guanine-N(7)-)-methyltransferase subunit WDR4 [Zootermopsis nevadensis]	3.426915232	3.457876862
Cluster-19019.1685	Leucine-rich PPR motif-containing protein, mitochondrial [Cryptotermes secundus]	3.479157294	3.513366251
Cluster-96.0	HSP12 [Dugesia japonica]	6.231151854	3.349339886
Cluster-19019.9994	chaperonin [Eurycantha calcarata]	3.22146244	3.222378986
Cluster-16257.1	hypothetical protein LSTR_LSTR006597 [Laodelphax striatellus]	3.477619785	6.108292342
Cluster-9625.0	BCL2/adenovirus E1B 19 kDa protein-interacting protein 3 [Cryptotermes secundus]	3.523690155	3.110951842
Cluster-15295.0	dynein light chain roadblock-type 2-like isoform X2 [Nilaparvata lugens]	3.02432167	3.756244649
Cluster-19019.5394	PREDICTED: RNA-directed DNA polymerase from mobile element jockey-like [Amyelois transitella]	3.856465686	3.241554426
Cluster-1547.0	catalase [Harpegnathos saltator]	8.829506781	3.066932372
Cluster-1960.0	catalase/peroxidase HPI [Thecamonas trahens ATCC 50062]	3.768642479	3.747055344
Cluster-366.0	hypothetical protein PBRA_008791 [Plasmodiophora brassicae]	3.112947503	4.547441904
Cluster-1801.0	glutathione peroxidase Hyr1, putative [Acanthamoeba castellanii str. Neff]	3.428980046	3.489727546
Cluster-432.0	PREDICTED: catalase-peroxidase-like, partial [Plutella xylostella]	2.862942963	4.974496207
Cluster-2997.0	PREDICTED: aromatic peroxygenase-like [Bemisia tabaci]	2.727990579	3.301107161
Cluster-327.0	catalase hydroperoxidase hpi, partial [Lasius niger]	2.703138557	2.726336056
Cluster-22555.2	PREDICTED: DDRGK domain-containing protein 1 [Aethina tumida]	2.703554051	3.067474867
Cluster-23143.0	sestrin homolog [Nilaparvata lugens]	2.747165867	2.549002137
Cluster-19019.7449	probable cytochrome P450 304a1 [Cryptotermes secundus]	2.81962926	8.433898659

Cluster-16386.0	probable cytochrome P450 305a1 [Cimex lectularius]	2.663530419	3.146370067
Cluster-5177.0	probable cytochrome P450 6a13 [Cryptotermes secundus]	3.749362084	4.088665445
Cluster-19019.10741	probable cytochrome P450 6a13 [Cryptotermes secundus]	3.492903305	2.564318879
Cluster-19019.10806	PREDICTED: probable cytochrome P450 49a1 [Bemisia tabaci]	2.601785565	5.294375572
Cluster-16236.0	forkhead box protein O [Laodelphax striatellus]	3.815835395	2.40532943
Cluster-16286.0	hypothetical protein LSTR_LSTR006652 [Laodelphax striatellus]	2.738401196	5.235641828
Cluster-16719.0	longitudinals lacking protein, isoforms A/B/D/L isoform X48 [Agrilus planipennis]	2.535698889	8.196487503
Cluster-18009.0	myelin transcription factor 1 isoform X3 [Bombyx mori]	2.626638433	4.213290857
Cluster-17520.0	TP53-regulated inhibitor of apoptosis 1-like [Cimex lectularius]	2.472633173	2.459486509
Cluster-17520.0	TP53-regulated inhibitor of apoptosis 1-like [Cimex lectularius]	3.172764228	2.997643283
Cluster-19019.4612	apoptosis-stimulating of p53 protein 1 isoform X3 [Cryptotermes secundus]	3.282427717	3.305658682
Cluster-19019.4612	apoptosis-stimulating of p53 protein 1 isoform X3 [Cryptotermes secundus]	2.457957877	2.501828753
Cluster-19019.6918	probable Bax inhibitor 1 [Nilaparvata lugens]	2.868965661	8.159142212
Cluster-19337.0	glutathione S-transferase 12 [Subpsaltria yangi]	2.4822405	5.142798959
Cluster-19019.3758	glutathione S-transferase [Apolygus lucorum]	3.896363427	2.680641499
Cluster-12039.0	microsomal glutathione S-transferase 1-like [Cimex lectularius]	2.387610888	4.168143015
Cluster-19019.3921	carboxylesterase E10 [Subpsaltria yangi]	2.344862132	2.650818069
Cluster-11905.0	carboxylesterase E14 [Subpsaltria yangi]	3.427894169	2.510944677
Cluster-19019.11184	carboxylesterase E2 [Subpsaltria yangi]	2.27873953	7.999271175
Cluster-1517.0	Superoxide dismutase [Mn], mitochondrial [Orchesella cincta]	2.937905807	2.223372407
Cluster-3522.0	Superoxide dismutase, copper/zinc binding domain [Pseudocohnilembus persalinus]	8.018320357	4.193177272
Cluster-19019.8037	superoxide dismutase [Cu-Zn]-like isoform X2 [Nilaparvata lugens]	2.327720478	2.807694341
Cluster-1547.0	catalase [Harpegnathos saltator]	2.260672037	3.23006518
Cluster-1960.0	catalase/peroxidase HPI [Thecamonas trahens ATCC 50062]	2.249228102	2.212229751
Cluster-5986.0	PREDICTED: catalase-peroxidase-like, partial [Plutella xylostella]	2.178530263	2.744760353
Cluster-1801.0	glutathione peroxidase Hyr1, putative [Acanthamoeba castellanii str. Neff]	2.292438061	4.251778333
Cluster-27.0	heat shock protein 70 [Leishmania braziliensis]	7.881373167	5.624837707
Cluster-14733.0	heat shock protein 23 [Ceratitis capitata]	2.209893926	2.151951734
Cluster-2541.0	heat shock protein 60 [Galeruca daurica]	2.401166794	2.962442317
Cluster-1236.0	heat shock protein [Capsaspora owczarzaki ATCC 30864]	2.427349941	3.540891713
Cluster-1166.0	heat shock protein 90, partial [Neobodo saliens]	2.405954851	7.912160689
Cluster-19019.8357	glutathione peroxidase 3-like [Nilaparvata lugens]	2.855128618	7.912160689
Cluster-15386.0	Inhibitor of growth protein 4 [Blattella germanica]	2.063340107	7.912160689

Cluster-11719.0	UDP-N-acetylglucosamine transferase subunit ALG14 homolog [Nilaparvata lugens]	2.40412526	7.86655019
Cluster-19019.13282	UDP-glucuronosyltransferase 2B33-like [Nilaparvata lugens]	2.548765702	4.140533965
Cluster-19019.1999	multidrug resistance-associated protein 5-like isoform X7 [Nilaparvata lugens]	2.005707692	2.866989409
Cluster-19019.12500	death-associated protein 1 [Athalia rosae]	2.46583294	2.139127097
Cluster-2308.0	membrane beta-glucosidase 1 [Physarum polycephalum]	11.66324693	12.77840303
